# Near-zero Fluoroscopic Approach for Laser Balloon Pulmonary Vein Isolation Ablation: A Case Study

**DOI:** 10.19102/icrm.2020.110402

**Published:** 2020-04-15

**Authors:** Henry D. Huang, Nicholas Serafini, Jason Rodriguez, Parikshit S. Sharma, Kousik Krishnan, Richard G. Trohman

**Affiliations:** ^1^Division of Internal Medicine, Section of Cardiology, Rush University Medical Center, Chicago, IL, USA

**Keywords:** Atrial fibrillation, fluoroless ablation, laser balloon, pulmonary vein isolation, zero fluoroscopy

## Abstract

Fluoroscopy remains a cornerstone imaging modality for catheter placement and positioning in electrophysiology device and ablation procedures. However, efforts are being made to reduce the cumulative exposure to radiation in the patient and physician alike. We present the case of a 59-year-old male patient with hypertension, chronic kidney disease, and paroxysmal atrial fibrillation who underwent successful near-fluoroless laser balloon (LB) pulmonary vein isolation (PVI) ablation. Though this case demonstrates the usage of a novel protocol for near-fluoroless LB ablation that resulted in successful, uncomplicated acute PVI, the feasibility and safety of this technique should be validated in a larger series or prospective comparative study.

## Case presentation

A 59-year-old male with hypertension, chronic kidney disease, and paroxysmal atrial fibrillation (AF) was referred for catheter ablation due to an increasing frequency of symptomatic AF episodes over the past year despite antiarrhythmic drug (ADD) therapy. After discussion of the risks and benefits of catheter ablation and different modalities for pulmonary vein (PV) isolation (PVI), the patient opted to undergo visually guided laser balloon (LB) ablation. A baseline transthoracic echocardiogram revealed normal left ventricular ejection fraction (55%) and left atrial (LA) volume index (32.1 mL/min).

### Procedure details

**Figure 1:**
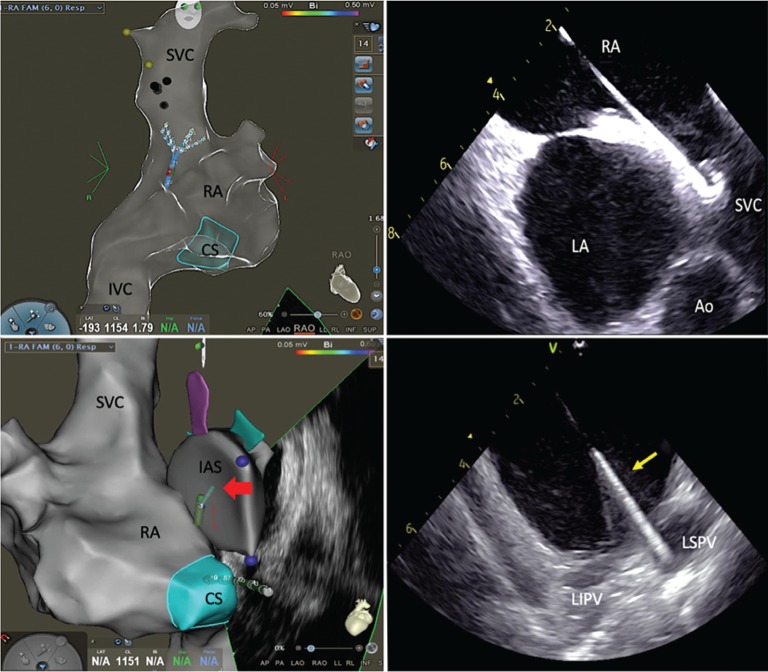
**A:** 3D geometry created of the RA, CS ostium, SVC, and IVC with multispline mapping catheter and 3D mapping system. Locations with PN capture during pacing are tagged on the wall of the SVC. **B:** View of the RA–SVC junction during advancement of the 0.032-in guidewire obtained with posterior flexion on the ICE catheter in the RA. **C:** EAM- and ICE-guided placement of the transseptal apparatus on the interatrial septum. **D:** Exchange wire placed within the LSPV on ICE. Ao: aorta; IAS: interatrial septum; TS: transseptal.

After informed consent was obtained, the patient was brought to the electrophysiology (EP) laboratory and placed under general anesthesia for the procedure. Percutaneous femoral venous access was obtained using ultrasound guidance and modified Seldinger technique. An 8-French (Fr) intracardiac echocardiography (ICE) catheter (AcuNav™; Biosense Webster, Diamond Bar, CA, USA) was advanced into the right atrium (RA) and a three-dimensional (3D) electroanatomic shell was created of the RA, LA, superior vena cava (SVC), and coronary sinus (CS) ostium and integrated into the CARTO^®^ 3 mapping system (CARTOSOUND^®^; Biosense Webster, Diamond Bar, CA, USA). A five-spline multielectrode mapping catheter (Pentaray^®^; Biosense Webster, Diamond Bar, CA, USA) was advanced to the inferior vena cava (IVC)–RA junction and a 3D fast anatomic map of the RA cavity, ostium, proximal body of the CS, and SVC was created. A detailed map of the course of the right phrenic nerve (PN) was also developed during high-output pacing from the mapping catheter’s distal poles beginning at the right innominate artery–SVC junction and sweeping down to the SVC-RA junction **(Figure 1A)**. Next a 7-Fr decapolar catheter (Biosense Webster, Diamond Bar, CA, USA) was positioned in the CS using electroanatomic mapping (EAM) guidance and previously acquired anatomy as visual landmarks.

The multispline mapping catheter was then exchanged for a long 0.032-in J-wire, which was positioned in the SVC under ICE visualization (ie, high in the RA with a posterior tilt) without the use of fluoroscopy **(Figure 1B)**. A 63-cm-long SL1 sheath (Abbott Laboratories, Chicago, IL, USA) was advanced over the long 0.032-in J-wire into the SVC under ICE visualization. The 0.032-in J-wire was then exchanged for a BRK-1 transseptal needle (Abbott Laboratories, Chicago, IL, USA), and brief fluoroscopy was used to confirm that the tip of the transseptal needle did not extend beyond the tip of the SL1 dilator. The proximal end of the BRK-1 needle apparatus was connected to alligator cables (Abbott Laboratories, Chicago, IL, USA) and a pin box to facilitate live tracking of the needle tip on the CARTO^®^ 3 mapping system (CARTOSOUND^®^; Biosense Webster, Diamond Bar, CA, USA). Under EAM guidance, the transseptal assembly was pulled down from the SVC onto the fossa ovalis **(Figure 1C)**. The presence of interatrial septal tenting was visually confirmed on ICE imaging in the plane of the PVs. Successful puncture of the interatrial septum with the BRK-1 needle was confirmed on ICE by the presence of bubbles within the LA, and the SL1 was advanced into the LA. The BRK-1/dilator assembly was exchanged for the multispline mapping catheter, and a 3D map of the LA and PVs was created.

### Laser balloon ablation

**Figure 2:**
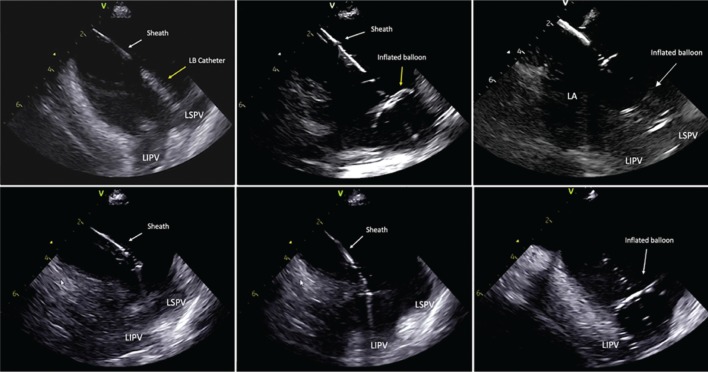
**A:** LB catheter advanced directly into the LSPV under ICE visualization. **B:** LB inflated until the proximal side of the balloon is seen occluding the entire LSPV antrum. **C:** The inflated LB was pulled back into the LA cavity prior to repositioning in the LSPV for better occlusion. **D:** After finishing the ablation of the LSPV, the LB catheter was pulled back into the sheath. **E:** The sheath–catheter assembly is flexed and slightly clocked under ICE visualization until it was pointing toward the LIPV. **F:** Cannulation and inflation of the LB showed antral occlusion of the LIPV with the balloon.

**Figure 3:**
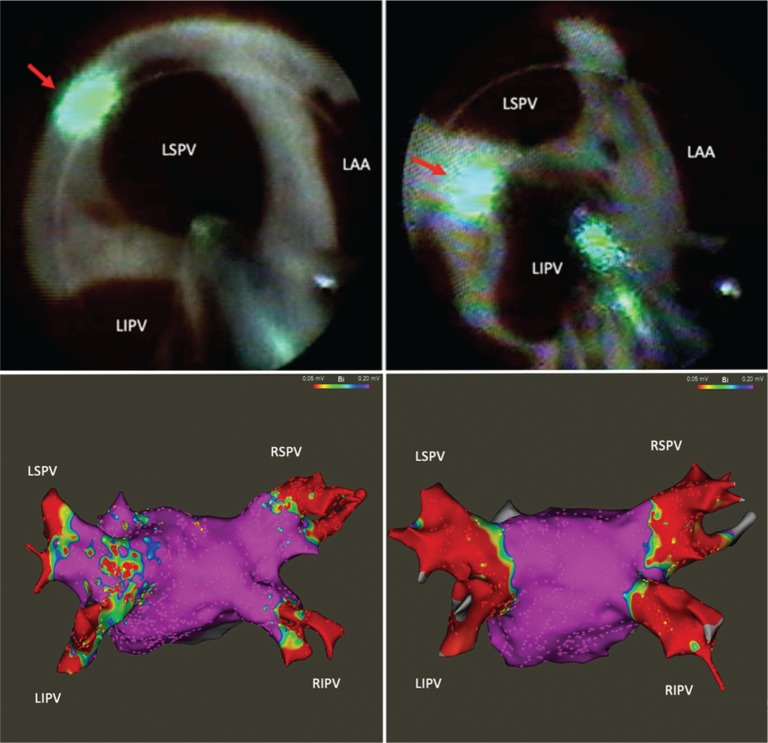
**A:** LB catheter with the tip of balloon positioned in the lower branch of the LSPV. The red arrow shows the green illuminated area from the beam aimed on the superoposterior wall. **B:** LB catheter with the tip of the balloon positioned in an upper branch of the LIPV. The red arrow shows the green illuminated area from the beam aimed on the posterior carina. **C:** 3D voltage map of the posterior LA and pulmonary veins prior to laser balloon ablation. **D:** Voltage map of the posterior LA and pulmonary veins acquired following laser balloon ablation. LAA: left atrial appendage.

The multispline mapping catheter was removed, and an Amplatz super stiff guidewire (Boston Scientific, Natick, MA, USA) was advanced through the SL1 sheath and placed into the left superior PV (LSPV) under ICE imaging **(Figure 1D)**. While maintaining constant ICE visualization of this wire in the LSPV, the SL1 sheath was exchanged for a 12-Fr steerable sheath-dilator assembly (CardioFocus, Marlborough, MA, USA). The LB catheter (HeartLight Excalibur™; CardioFocus, Marlborough, MA, USA) was advanced through the 12-Fr sheath and into the LA cavity. Under ICE visualization, the soft, atraumatic tip of the LB catheter was directly advanced into an upper branch of the LSPV without tactile resistance **(Figure 2A)**. The catheter’s balloon was then manually inflated under endoscopic and ICE visualization **(Figure 2B)**. The ideal antral position of the equatorial surface of the LB was confirmed on ICE by the presence of the balloon’s proximal end within the LA cavity and by endoscopic landmark visualization **(Figure 3A)**. If the position of the catheter’s distal end interferes with obtaining coaxial alignment of the balloon with the PV ostium, the LB can be pulled back into the LA cavity while still inflated **(Figure 2C)** and the PV can be reengaged by gently pushing forward with the LB while viewing on ICE until the tip is advanced into a different side branch.

Following delivery of a circumferential lesion set around the LSPV antrum, the LB was deflated and withdrawn from the LSPV. Under ICE visualization, the sheath was deflected until the catheter tip was pointing directly toward the ostium of the left inferior PV (LIPV) **(Figures 2D and 2E)**. The LB catheter was advanced into the LIPV and inflated until antral balloon contact was confirmed with endoscopic and ICE visualization **(Figure 2F)**. A similar circumferential laser lesion set was created to isolate the LIPV **(Figure 3B)**.

**Figure 4:**
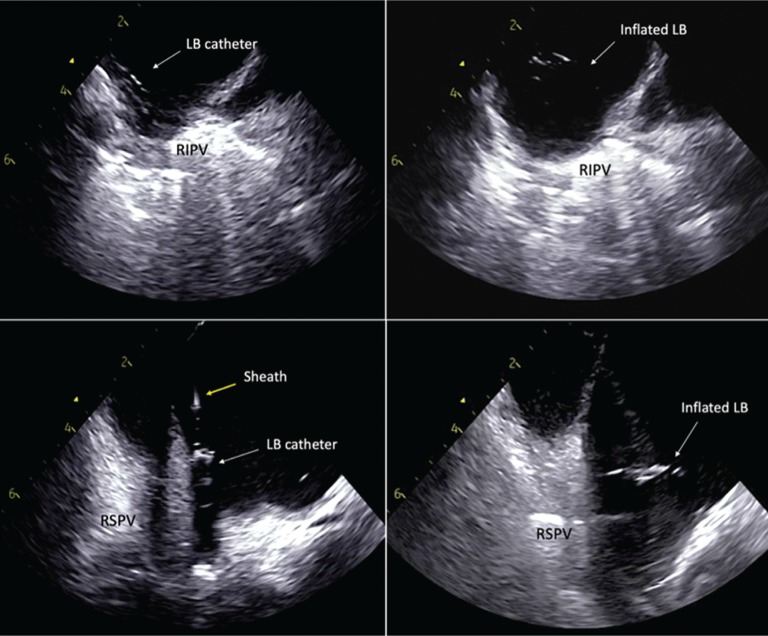
**A and B:** LB catheter advanced into the RIPV under ICE visualization after the “hockey-stick” maneuver and inflated to cover the antrum of the RIPV for ablation. **C and D:** After removing the LB catheter from the RIPV and adding posterior flexion to the ICE catheter, the sheath is undeflected and slightly clockwise rotated until the sheath–catheter assembly is seen in the same plane as the RSPV on ICE. The LB catheter is then advanced into the PV and inflated until the balloon is visualized occluding the antrum of a very large RSPV.

To cannulate the right inferior PV (RIPV) under ICE visualization, we utilized a “hockey-stick” maneuver. First, the LB catheter was withdrawn until just the atraumatic tip was exposed beyond the 12-Fr sheath. While still facing the left PVs, we maximally deflected the sheath and withdrew the assembly toward the interatrial septum while maintaining a curve on the sheath. To avoid damage to the LA posterior wall during this maneuver, brief fluoroscopy was used to reconfirm that the LB catheter’s soft tip remained beyond the sheath prior to RIPV cannulation. Using ICE, the 12-Fr sheath was rotated clockwise until the sheath–catheter assembly was visualized in the same plane as the RIPV **(Figure 4A)**. Following cannulation of the RIPV and inflation of the LB using the aforementioned steps, the decapolar catheter was removed from the CS and aligned with previously tagged sites of PN capture. While pacing at a suprathreshold output, an antral circumferential RIPV lesion set was created while simultaneously limiting the likelihood of PN injury by monitoring the compound motor action potential amplitude and manually assessing diaphragmatic movement.

For cannulation of the right superior PV (RSPV), the ICE catheter was advanced superiorly with slight posterior deflection until the vein was visualized. After withdrawing the LB catheter from the RIPV, deflection on the 12-Fr sheath was released and the sheath–catheter assembly was slowly clocked until the LB catheter was seen in the same plane as the RSPV on ICE **(Figure 4C)**. The LB catheter was then advanced into the RSPV and circumferential ablation was performed while monitoring for PN injury as described above.

After LB ablation of all four PVs was visually completed, the LB catheter was exchanged for a multielectrode mapping catheter and a postablation high-density voltage map was created of the LA and PVs, confirming entrance block **(Figures 3C and 2D)**. Pacing from the multielectrode catheter positioned at the ostium of each PV was used to demonstrate exit block. The LB catheter–sheath assembly was then removed from the LA. After checking the final activated clotting time, protamine was administered intravenously and all remaining sheaths and catheters were removed. Hemostasis was obtained with manual pressure.

There were no complications during the procedure. The final ICE images of the pericardial space revealed no evidence of pericardial effusion. The patient resumed his direct oral anticoagulation regimen the same evening and was discharged from the hospital the following day. His AAD was discontinued three months after ablation and he experienced no recurrence of AF during eight months of follow-up.

### Procedure duration and fluoroscopy time

Overall, the total procedure time was 146 minutes and the LA dwell time was 59 minutes. The fluoroscopy setting was 7.5 frames per second. The fluoroscopy time required to obtain transseptal access was 0.3 minutes. The total fluoroscopy time in the LA to accomplish PVI and confirm bidirectional block of all four PVs was 0.2 minutes. Thus, the total procedural fluoroscopy time was 0.5 minutes.

## Discussion

Fluoroscopy remains a cornerstone imaging modality for catheter placement and positioning in electrophysiology (EP) device and ablation procedures. However, cardiovascular procedures are believed to account for approximately 40% of the effective radiation dose in the general population.^1^ Although overall fluoroscopy use during EP ablation procedures has decreased in the past decade with the adoption of 3D EAM systems and ICE imaging, the hazards of cumulative ionizing radiation exposure remain a concern for both operators and patients. This concern is increasingly relevant as the volume and complexity of cardiac EP procedures increase.^2^

Patients referred for AF catheter ablation are at an especially increased risk of high radiation exposure during treatment. In large catheter ablation trials,^3,4^ the average fluoroscopy time for PVI-only AF ablation procedures varied between 20 minutes and 60 minutes. Single-case exposure during AF catheter ablation is estimated to be roughly equivalent to undergoing more than 800 chest radiographs.^1^ Furthermore, many patients who undergo balloon-based PVI ablation modalities receive additional radiation exposure from preprocedure contrast tomography imaging to evaluate PV anatomy. Patients may also require redo ablation procedures after their index PVI procedure for recurrent symptomatic AF episodes, further increasing their radiation exposure.

In a multicenter randomized pivotal trial comparing LB–PVI to radiofrequency–PVI, the mean fluoroscopy time in the LB group was 35.6 minutes ± 18.2 minutes.^5^ Although subsequent LB studies with experienced users have demonstrated a reduction in fluoroscopy usage per case, the mean fluoroscopy time in LB–PVI groups has remained between 12 minutes and 28 minutes.^6–8^ Fluoroless AF ablation has been demonstrated to be feasible and safe in comparison with conventional AF ablation approaches, but most studies to date have examined point-by-point radiofrequency ablation–PVI, which allows for the integration of most aspects of the procedure with 3D mapping system guidance.^9,10^ Currently, data regarding the feasibility and safety of low-fluoroscopy approaches for balloon-based PVI modalities are limited.^11–13^ In a small cohort study, Demo et al. demonstrated the potential feasibility of a zero-fluoroscopic approach in five consecutive patients who underwent successful cryoballoon ablation without the use of contrast venography.^13^

Based on our experience, visually guided LB ablation offers several advantages over CB ablation for minimizing fluoroscopy usage during PVI including direct endoscopic visualization of PVs and other anatomic landmarks. The use of tapered, compliant balloons with adjustable sizes (up to 38 mm), which are better suited for the cannulation of small veins and large ovoid-shaped or common ostium PVs, may decrease the need for balloon repositioning; and in PV anatomy, where a complete 360-degree seal is difficult to achieve, such has the ability to deliver point-by-point lesions without compromising the quality of energy delivery as long as myocardial tissue is visualized. Meanwhile, the CB technique offers simultaneous PV electrogram monitoring during ablation and easier integration with 3D mapping systems because of its circular mapping catheter, whereas the current-generation LB catheter lacks electrodes for real-time electrogram (EGM) monitoring. Therefore, a second transseptal puncture and a diagnostic catheter are required if simultaneous PV EGM monitoring during LB ablation is desired.

In the largest series studying long-term outcomes of zero-fluoroscopy ablation, the authors demonstrated that zero-fluoroscopy use during ablation procedures could be successfully accomplished for all types of ablation cases except patients who underwent AF ablation (presumably attributed to the risk of complications during transseptal access and catheter manipulation within the LA).

To our knowledge, this is the first reported case of near-fluoroless LB ablation resulting in successful, uncomplicated acute PVI. However, this is a single case study and the feasibility and safety of this technique should be validated in a larger series or a prospective comparative study. One potential challenge for adopting this near-zero fluoroscopy technique is the learning curve associated with relying on ICE and endoscopic visualization as primary imaging modalities during PVI procedures. Further, in clinical practices where ICE and 3D mapping are not routinely employed during PVI, the benefits of fluoroscopy reduction will need to be weighed against the cost of additional equipment usage and the learning curve required for the operator to master additional techniques required. Although we do believe that zero-fluoroscopy usage is likely achievable for LB PVI, such an approach may be associated with a steeper learning curve and—at least, initially—possibly a higher risk of procedural complications.

### Summary

This is the first case to describe the successful use of a near-zero fluoroscopic approach for LB ablation to achieve PVI.
